# Network Modeling and Energy-Efficiency Optimization for Advanced Machine-to-Machine Sensor Networks

**DOI:** 10.3390/s121114851

**Published:** 2012-11-05

**Authors:** Sungmo Jung, Jong Hyun Kim, Seoksoo Kim

**Affiliations:** 1 Department of Multimedia, Hannam University, Daejeon 306-791, Korea; E-Mail: sungmoj@gmail.com; 2 Electronics and Telecommunications Research Institute, Daejeon 305-700, Korea; E-Mail: jhk@etri.re.kr

**Keywords:** clustering algorithm, lifespan, machine to machine, multiple radio interfaces, network modeling, wireless sensor networks

## Abstract

Wireless machine-to-machine sensor networks with multiple radio interfaces are expected to have several advantages, including high spatial scalability, low event detection latency, and low energy consumption. Here, we propose a network model design method involving network approximation and an optimized multi-tiered clustering algorithm that maximizes node lifespan by minimizing energy consumption in a non-uniformly distributed network. Simulation results show that the cluster scales and network parameters determined with the proposed method facilitate a more efficient performance compared to existing methods.

## 1. Introduction

In machine-to-machine (M2M) sensor networks, individual nodes may be stationed in a non-uniform manner, depending on the topography of the environment and the specific application. In such cases, it is particularly important to form uniform clusters in the network. To this end, advanced sensor nodes with multiple radio interfaces are expected to have several advantages, including high spatial scalability, low event detection latency, and low energy consumption [1]. However, it is also essential to develop methods to maximize network lifespan by minimizing the energy consumption of sensor nodes.

Several hierarchical clustering methods (LEACH, PEGASIS, TEEN, *etc.*) have been suggested for increasing network energy efficiency, and a variety of cluster head (CH) election methods have been studied. In addition, a few advanced methods (M-LEACH, HEED, EEHC, *etc.*) have been proposed for mobile nodes such as those in an M2M sensor network [2]. However, these methods have several limitations. First, efficient cluster formation becomes more difficult with increasing network scale. Second, if the sensor nodes are not evenly distributed in the network environment, uniform cluster formation is impossible.

Here, we propose a network modeling method and an energy-efficient clustering algorithm for advanced M2M sensor networks. We also present the results of simulations in which the proposed method is compared with existing methods.

## 2. Related Research

### 2.1. Low Energy Adaptive Clustering Hierarchy

The LEACH [3] protocol is an energy-efficient protocol that extends system lifetime. LEACH is designed for sensor networks where an end-user wants to remotely monitor the environment. In such a situation, the data from the individual nodes must be sent to a central base station (BS), often located far from the sensor network, through which the end-user can access the data. There are several desirable properties for protocols on these networks:

1. Use 100 s–1,000 s of nodes
2. Maximize system lifetime
3. Maximize network coverage
4. Use uniform, battery-operated nodes

Conventional network protocols, such as direct transmission, minimum transmission energy, multi-hop routing, and clustering all have drawbacks that don't allow them to achieve all the desirable properties. LEACH includes distributed cluster formation, local processing to reduce global communication, and randomized rotation of the CHs. These features allow LEACH to achieve the desired objectives.

### 2.2. Power-Efficient Gathering in Sensor Information Systems

The PEGASIS [4] protocol is a chain-based protocol. In general, the PEGASIS protocol presents twice or more performance in comparison with the LEACH one. However, PEGASIS causes redundant data transmissions since one of nodes on the chain is selected as the head node, regardless of the BSs location.

### 2.3. Threshold Sensitive Energy Efficient Sensor Network

The TEEN [5] protocol is a hierarchical clustering protocol, which groups sensors into clusters with each led by a CH. The sensors within a cluster report their sensed data to their CH. The CH sends aggregated data to higher level CH until the data reaches the sink. Thus, the sensor network architecture in TEEN is based on a hierarchical grouping where closer nodes form clusters and this process goes on the second level until the BS (Sink) is reached. TEEN is useful for applications where the users can control a trade-off between energy efficiency, data accuracy, and response time dynamically. TEEN uses a data-centric method with hierarchical approach.

### 2.4. Multihop-LEACH

A LEACH CH always transmits data directly to the BS, regardless of the distance between them. To reduce energy, the M-LEACH [6] protocol, another variant on the LEACH theme, chooses an optimal path between a CH and the BS through other CHs. These CHs transmit data to the CH which is nearest to BS. Finally, this CH sends data to BS. M-LEACH is almost the same as LEACH, but the difference is that the communication mode in M-LEACH is multi-hop between CHs and BS. M-LEACH has better energy efficiency than LEACH in many cases.

### 2.5. Hybrid Energy-Efficient Distributed Clustering Approach

HEED [7] is a hybrid: CHs are probabilistically selected based on their residual energy, and nodes join clusters such that communication cost is minimized. HEED parameters, such as the minimum selection probability and network operation interval, can be easily tuned to optimize resource usage according to the network density and application requirements. HEED achieves a connected multi-hop inter-cluster network when a specified density model and a specified relation between cluster range and transmission range hold.

### 2.6. Energy Efficient Heterogeneous Clustering Approach

EEHC [8] is a distributed randomized clustering algorithm that maximizes the lifetime of a network with a large number of sensor nodes. EEHC organizes the sensors in a network into clusters with a hierarchy of CHs. The CHs collect the information from the sensor nodes within their clusters and send an aggregated report through the hierarchy of CHs to the BS. EEHC assumes that communication environment is contention and error free. The energy consumed in network will depend on: (i) the probabilities of each sensor node becoming a CH at each level in the hierarchy and (ii) the maximum number of hops allowed between one cluster node and its CH. The optimal clustering parameters are obtained through hierarchical clustering to minimize the total energy consumption in the network. However, CHs in hierarchical model consume relatively more energy than other sensor nodes because CHs have more loads to handle. Hence, CHs may run out of their energy faster than other sensor nodes. Thus, EEHC can be run periodically for load balancing or triggered as the energy levels of the CHs fall below a certain threshold.

## 3. Network Model Design and Energy-Efficiency Optimization

### 3.1. Uniform Network Model

**Figure 1.**
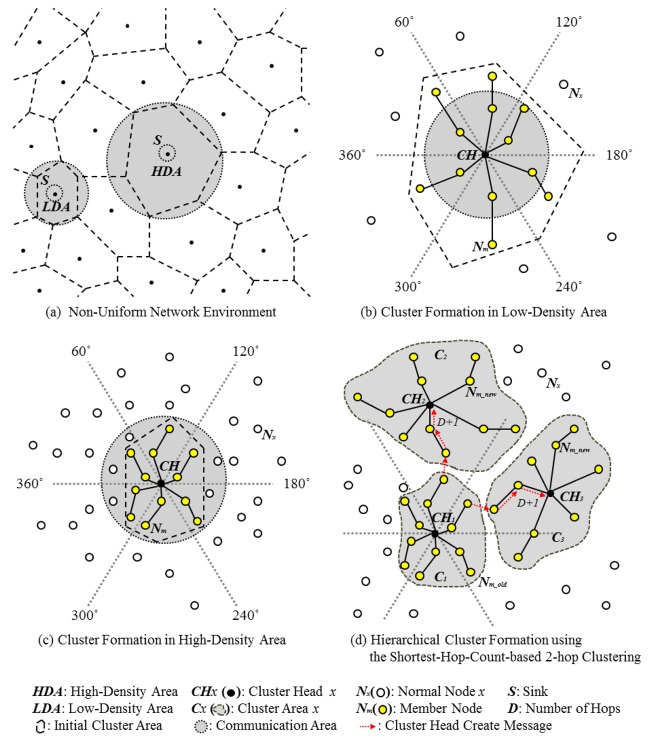
Hierarchical uniform cluster formation. (**a**) Non-uniform network environment. (**b**) Cluster formation in Low-Density Area. (**c**) Cluster formation in High-Density Area. (**d**) Hierarchical cluster formation using the shortest-hop-count-based clustering.

Consider a non-uniform network, such as the one shown in Figure 1(a). The network is divided into sections centered around sink nodes to show the cluster density. Cluster formation in the low- and high-density areas of this network occurs as shown in Figure 1(b,c), respectively. In these figures, the CH elects member nodes by using a logical-hop-count range and the shortest hop count in the 360° range. Figure 1(d) shows the result of hierarchical cluster formation. Shortest-hop-count-based 2-hop clustering was used at 60° intervals in order to generate the circles. In the angle range, the initial multi-hop cluster (*C_1_*) is created using the shortest hop count. The terminal node sends a CH create request message to nodes within *D*+*1* hops from itself. *CH_2_* and *CH_3_* receive this request message as they are within *D*+*1* hops from *C_1_*'s terminal node.

Thus, they form the new CHs of clusters *C_2_* and *C_3_*. However, some nodes receive duplicate cluster join messages. Such nodes must decide which cluster to join on the basis of the communication cost. Therefore, the network model should be constructed as a multi-tiered structure: the first tier collects intracluster data and the second tier collects information on CHs; the second tier begins from the sink node and extends toward the interior of the cluster.

**Figure 2.**
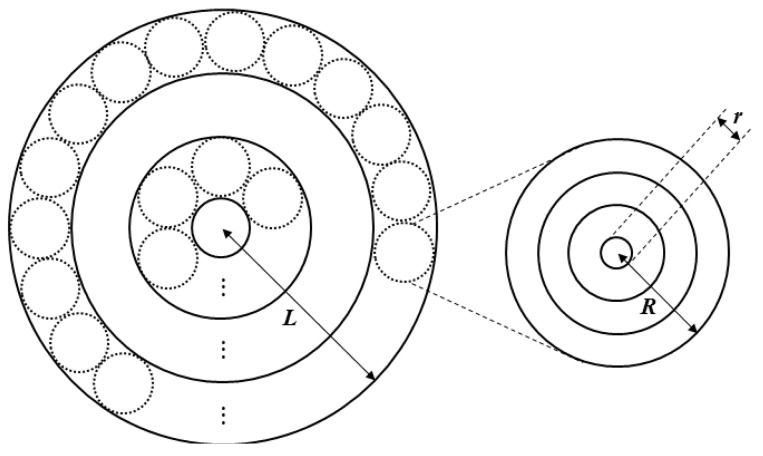
Network approximation model.

The scale and topography of each cluster differ since the node density differs. Thus, we propose the network approximation model shown in Figure 2. All networks are approximated by a multi-tiered network, shown by a circle of radius *L*, and the constructed clusters are represented by the small circles of radius *R*. The network model has a donut-shaped ring structure, which is convenient for forming a set of clusters located at the same distance from the sink node.

Both the first- and second-tier clusters are approximated by the same method, and data transmission begins from the outermost ring and progresses toward the interior in a gradual manner. Ultimately, the data are passed to the sink node. At the first tier, the transmission distance is less than that at the second tier and so a relatively small amount of data is transmitted. At the second tier, in contrast, a relatively large amount of data is transmitted. Multiple radio interfaces are used for each tier to allow energy-efficient data transmission. Specifically, the first tier uses a low-speed radio interface and the second tier uses a high-speed radio interface.

### 3.2. Use of Clustering Algorithm to Optimize Energy Efficiency

The clustering algorithm proposed here allows the formation of clusters of similar scales by using the network approximation modeling algorithm shown in Algorithm 1.

**Algorithm 1.** Proposed clustering algorithm to optimize energy efficiency.*// Assign Initial-Value*# own.ID ← own ID# src.ID ← received own.ID# sink ← first sink node ID# sink.toward ← src.ID# provisional.CH ← true# msg.odr.RSSI, own.msg.odr.RSSI ← 0# thrhold.RSSI ← ρ# bc.elect.msg ← src.ID, hop.count, msg.odr.RSSI# own.elect.msg ← own.ID, own.hop.count, own.msg.odr.RSSIDO (hop.count = 0)*// Broadcast of CH Election Order Message from the First Sink-node* IF (sink! = null)  msg.odr ← sink, hop.count  broadcast msg.odr  ELSE   BREAK ENDIF *// Cluster Head Election and Elected Message Broadcasting* IF (get msg.odr(src.ID, hop.count)) THEN  own.msg.odr.RSSI ← RSSI value of msg.odr  ELSE IF (msg.odr.RSSI < thrhold.RSSI ‖ own.hop.count < hop.count ‖ msg.odr.RSSI < own.msg.odr.RSSI)   own.hop.count ← hop.count   own.msg.odr.RSSI ← msg.odr.RSSI   generate sink.toward   bc.elect.msg ← own.elect.msg   broadcast bc.elect.msg  ENDIF ENDIF *// Compare RSSI* IF (get bc.elect.msg) THEN  elect.msg.RSSI ← RSSI value of elect.msg  ELSE IF (elect.msg.RSSI < thrhold.RSSI ‖ own.hop.count < hop.count) IF (own.msg.odr.RSSI > msg.odr.RSSI) then    provisional.CH ← false    BREAK   ENDIF  ENDIF ENDIF *// Repeat Cluster Head Election* IF (provisional.CH = true) THEN  SET self AS NEW CH  hop.count ← hop.count++  broadcast msg.odr(own.ID, hop.count) ENDIFWHILE (own.hop.count = ∞)

The above algorithm begins broadcasting the CH election order message (*msg.odr*) from the initial sink node. *msg.odr* includes the ID of the node that transmitted the message (*src.ID*) and the hop count (*hop.count*) from the sink node. The neighboring nodes receive *msg.odr* and record the message signal strength (*msg.odr.RSSI*). For the clusters, there is a specified threshold value for the received signal strength indication (RSSI) (*thrhold.RSSI*), and only sensor nodes within the transmission range of the circle can receive *msg.odr* for network approximation modeling. The nodes that receive *msg.odr* broadcast the CH election message (*elect.msg*), which includes *src.ID*, *hop.count*, and *msg.odr.RSSI*.

The nodes that receive *elect.msg* compare *own.hop.count* with the received *hop.count* message. If they are not equal, then the comparison result is ignored and messages are received only from nodes within the circle. Then, the number of nodes that receive *elect.msg* is greater than the number of nodes belonging to *S_x_* (set of nodes). The nodes that may belong to *S_x_* compare *own.msg.odr.RSSI* with *msg.odr.RSSI*, which is contained in each received *elect.msg*. If their own value is less than the received value, they elect themselves as CHs. An elected CH is within the angle range, and it is the node farthest from the sink node, among the nodes that belong to *S_x_*. The elected CHs increment *hop.count* by 1, broadcast *msg.odr*, and repeat the CH election process. There is a limit on the number of hops, and this limit is determined by the node density. Finally, they can assign their own cluster range and induct cluster members from the nodes within the range, as shown in Figure 1(d).

In the above-suggested clustering algorithm, there is an upper limit for the density since there is no CH cluster without a CH in the previous hop being in the range of the *thrhold.RSSI* of the CHs. In addition, there is also a lower limit to the density since the CH election process is repeated until there are no nodes left. The upper and lower limits of the cluster density *ρ* are as follows:

(1) $$\frac{4\sqrt{3}}{9}\leq \rho <\frac{4\sqrt{3}}{3}$$

### 3.3. Maximum Energy Consumption

To predict the network lifespan, we should determine the maximum energy consumption on the basis of the energy consumed by each node. To determine the maximum energy consumption, we define the (*i*,*j*)-th ring as the *i*-th ring in the first tier and the *j*-th ring in the second tier since each node is included in both tiers.

The first and second rings in the two tiers are candidates for the node with the maximum energy consumption. Thus, the maximum energy consumption range is decided by concentric rings consisting of the (*1*,*1*)-, (*1*,*2*)-, (*2*,*1*) and (*2*,*2*)-th rings.

The maximum energy consumption includes the *wake-up* energy, which is required to initialize the network from the sleep state. We propose that the maximum energy consumption rate is as follows:

(2) $$e_{\max\_\mathrm{avg}}=\sum_{i\in [1,2],j\in [1,2]}\left(E_{\text{time},i,j}\cdot \gamma +E_{\text{wake}\_\text{up}}\right)$$

The above formula can be used to compute the maximum average power (*e_max_avg_*) from the average energy consumption rate (*E_time,i,j_*), amount of data periodically handled by each node (*γ*), and the *wake-up* energy required to switch from the *idle mode* or *sleep mode* for each node (*E_wake-up_*).

The average energy required for transmitting and receiving data in the (*i*, *j*)-th ring (*E_time,i,j_*) is computed as follows:

(3) $$E_{\text{time},i,j}=\frac{DS_{Ft(s),i}\cdot P_{Ft(s),i}+DS_{Ft(r),i}\cdot P_{Ft(r),i}}{RA_{Ft,i}}+\frac{DS_{St(s),j}\cdot P_{St(s),j}+DS_{St(r),j}\cdot P_{St(r),j}}{RA_{St,j}}$$

where the subscript *Ft* denotes the first tier, *St* denotes the second tier, (*s*) indicates transmitting, and (*r*) indicates receiving. In addition, the data transmitted from the *x*-th ring in the *X*-th tier (*DS_Xt(s),x_*), energy consumption rate of the radio interface for transmission (*P_Xt(s),i_*), and ring width of each tier (*RA_Xt,x_*) are required.

The total energy consumption is obtained by multiplying the transmit and receive energies in each tier, and the average for each tier is the average of the values obtained by dividing the sum of the transmit and receive energies by the widths of the different rings.

Consider a network of radius *L* in where each tier has a constant number of rings *k* and the transmission and reception power are *P_Xs,x_* and *P_Xr.x_*, respectively. Then, it can be formulated as follows.

The data scale for transmitting and receiving is:

(4) $$DS_{Xs,x}=DS_{Xr,x-1}=\pi (L^{2}-{\left(2x-1\right)}^{2}\cdot R^{2}$$

The power consumption of the radio interface is:

(5) $$P_{Xs,x}=P_{Xr,x}=a_{x}+b_{x}\cdot {\left(2R\right)}^{C_{x}}$$

The ring width is:

(6) $$RA_{Xt,x}=8\pi \cdot k^{2}\cdot (x-1)$$

Here *a_x_*, *b_x_*, and *c_x_* are constants for the radio interface, *R* is the radius of the cluster, and *L* is given by *(2k* − *1)*·*R*. Each tier can be formed by the same method. However, the first tier does not have the same clusters as those in the second tier. Therefore, we can write *R* = *(2k* − *1)*·*r*.

The *wake-up* energy (*E_wake-up_*) is the sum of the energies of all tiers, and it can be computed as follows:

(7) $$E_{\text{wake}-\text{up}}=\frac{N_{\text{all}}\cdot W_{Ft}}{\pi L^{2}}+\frac{W_{St}}{\pi R^{2}}$$

where *N_all_* is the total number of sensor nodes (it is used to calculate the density of the first-tier sensors) and *W_Xt_* is the energy consumed during wake-up. The wake-up energy consumption of each tier is obtained by dividing *W_Xt_* by *πL^2^* and *πR^2^* for each cluster scale. In each tier, all nodes should wake up once for data transmission. Thus, the energy consumption is the same in all tiers of the network.

## 4. Performance Evaluation

In this paper, we assumed only one sink to compare the proposed method with existing methods (M-LEACH, HEED, and EEHC) by considering the clear parameters shown in Table 1.

In the simulation, we used CC2420 [9] for intracluster communication and IEEE 802.11g [10] for intercluster communication on the basis of technical standards for fair performance evaluation, the technical standards are obtained from experimental measurements performed in past studies [6–8].

**Figure 3.**
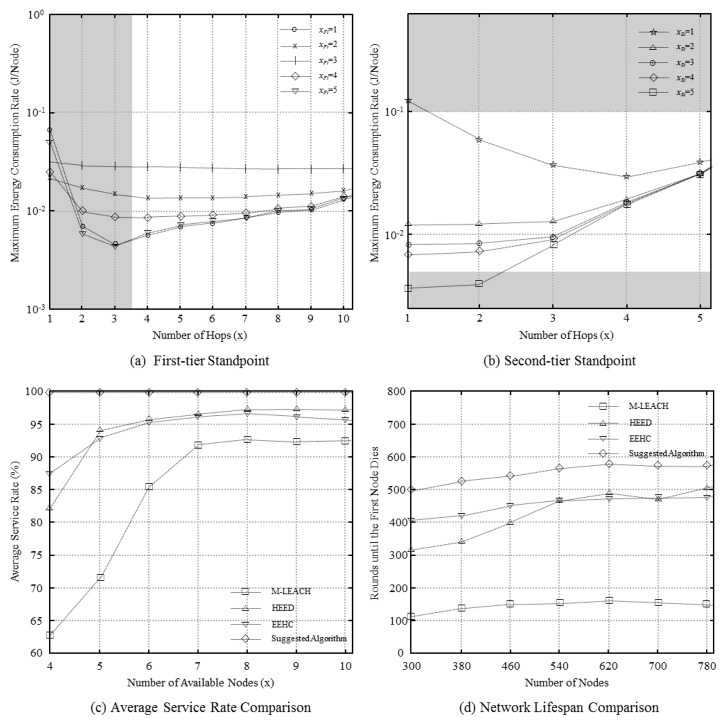
Performance evaluation. (**a**) First-tier standpoint; (**b**) Second-tier standpoint; (**c**) Average service rate comparison; (**d**) Network lifespan comparison.

The interface specifications are obtained by using the energy-consumption model of Equation (5) in the simulation. The results of the performance evaluation are shown in Figure 3.

In Figure 3(a,b), the maximum energy consumption rates for different hop counts are shown for each tier. The shaded areas are excluded since the transmission distance in these areas exceeds the maximum transmission distance, rendering them unsuitable for practical use. The results show that the lowest value of the maximum energy consumption rate corresponds to *x_Ft_* and *x_St_* values of 1 and 4, respectively; *x_Ft_* and *x_St_* are the energy-efficiency-optimized hop counts in the two tiers.

Figure 3(c) compares the number of available nodes as different nodes run out of energy. The results show that our algorithm has 7%, 3%, and 4% more available nodes than M-LEACH, HEED, and EEHC, respectively. In particular, we see that while the existing methods have lower energy efficiency at lower hop counts, this is not the case with our method.

Figure 3(d) compares the network lifespan for different numbers of nodes. For M-LEACH, we see that when the density of nodes increases with the number of nodes, the energy consumption of CHs increases. The increase in the energy consumption is because of clustering, which results from the large value of the absolute hop count. Consequently, the network lifespan decreases because of the energy consumption of CHs in relatively high-density areas. HEED and EEHC show relatively longer lifespans. However, the proposed method leads to about 17% greater lifespan.

## 5. Conclusions

We propose a network model design method involving network approximation and an optimized multi-tiered clustering algorithm that maximizes node lifespan by minimizing energy consumption in a nonuniformly distributed network. Simulation results show that cluster scales and network parameters determined with the proposed method lead to more efficient performance compared to existing methods. Based on this research, therefore, we plan to devise methods to maintain uniform clusters in network environments with frequently changing topology.

## Figures and Tables

**Table 1.** Simulation parameters.

**Description**	**Value**
Network size	500 m/L
Number of nodes	5,000
Data aggregation energy	5 nJ/bit/report
Initial energy	2.5 J
Energy consumed for short distance transmission	10 pJ/bit
Energy consumed for long distance transmission	0.0015 pJ/bit
Energy consumed to send or receive a signal	50 nJ/bit
Wake-up energy (CC2420 for 1st tier)	0.0347 mJ
Wake-up energy (IEEE 802.11g for 2nd tier)	5 mJ
Maximum transmission distance (CC2420)	60 m
Maximum transmission distance (IEEE 802.11 g)	100 m
